# Potential Role of Moesin in Regulating Mast Cell Secretion

**DOI:** 10.3390/ijms241512081

**Published:** 2023-07-28

**Authors:** Theoharis C. Theoharides, Duraisamy Kempuraj

**Affiliations:** 1 Institute for Neuro-Immune Medicine, Dr. Kiran C. Patel College of Osteopathic Medicine, Nova Southeastern University, Fort Lauderdale, FL 33328, USA; kduraisa@nova.edu; 2 Laboratory of Molecular Immunopharmacology and Drug Discovery, Department of Immunology, Tufts University School of Medicine, Boston, MA 02111, USA

**Keywords:** ERMs, flavonoids, luteolin, mast cells, mediators, moesin, phosphorylation, secretion, SNAREs, SNAPs, tryptase

## Abstract

Mast cells have existed for millions of years in species that never suffer from allergic reactions. Hence, in addition to allergies, mast cells can play a critical role in homeostasis and inflammation via secretion of numerous vasoactive, pro-inflammatory and neuro-sensitizing mediators. Secretion may utilize different modes that involve the cytoskeleton, but our understanding of the molecular mechanisms regulating secretion is still not well understood. The Ezrin/Radixin/Moesin (ERM) family of proteins is involved in linking cell surface-initiated signaling to the actin cytoskeleton. However, how ERMs may regulate secretion from mast cells is still poorly understood. ERMs contain two functional domains connected through a long α-helix region, the N-terminal FERM (band 4.1 protein-ERM) domain and the C-terminal ERM association domain (C-ERMAD). The FERM domain and the C-ERMAD can bind to each other in a head-to-tail manner, leading to a closed/inactive conformation. Typically, phosphorylation on the C-terminus Thr has been associated with the activation of ERMs, including secretion from macrophages and platelets. It has previously been shown that the ability of the so-called mast cell “stabilizer” disodium cromoglycate (cromolyn) to inhibit secretion from rat mast cells closely paralleled the phosphorylation of a 78 kDa protein, which was subsequently shown to be moesin, a member of ERMs. Interestingly, the phosphorylation of moesin during the inhibition of mast cell secretion was on the N-terminal Ser56/74 and Thr66 residues. This phosphorylation pattern could lock moesin in its inactive state and render it inaccessible to binding to the Soluble NSF attachment protein receptors (SNAREs) and synaptosomal-associated proteins (SNAPs) critical for exocytosis. Using confocal microscopic imaging, we showed moesin was found to colocalize with actin and cluster around secretory granules during inhibition of secretion. In conclusion, the phosphorylation pattern and localization of moesin may be important in the regulation of mast cell secretion and could be targeted for the development of effective inhibitors of secretion of allergic and inflammatory mediators from mast cells.

## 1. Introduction

Mast cells are specialized bone marrow-derived cells that play an important role in health [1] and in allergies [2,3,4,5,6,7,8,9,10,11,12] but also in innate and in adaptive immune processes [13,14,15,16], antigen presentation [16,17], regulation of T-cell responses [18,19,20], autoimmunity [21] and inflammation [10,22,23,24,25] in response to allergic and immunologic stress [4,26,27] but also non-allergic stress and toxic stimuli [10,28]. Mast cells are increased in number and are more reactive in mastocytosis [26] and mast cell activation syndrome (MCAS) [26,29,30], but they can also participate in other disorders [4,10,31,32,33], including neurotrauma, neuroinflammatory and neurodegenerative diseases [34,35,36].

## 2. Mast Cell Mediators and Mechanisms of Secretion

Mast cells are located in all tissues at the interface with the external environment [37] such as eyes, nose, lungs, skin and gastrointestinal tract. However, perivascular mast cells also sense the blood vessel lumen by extending filopodia through endothelial gaps and binding circulating immunoglobulin E (IgE) [38]. Mast cells are well known for their involvement in allergic and anaphylactic reactions via activation of the high-affinity surface receptor for IgE (FcεRI). Multivalent allergen binding leads to aggregation of FcεRI, leading to an influx of calcium ions, thus initiating a cascade of downstream events that involve phosphorylation of phosphatidyl inositol (IP3) and various Tyr kinases [39,40,41,42]. In addition to allergens, mast cells are also stimulated by a variety of triggers that include drugs, foods, pathogens and “danger signals” [26], as well as certain neuropeptides, especially substance P (SP) [43], via activation of their high-affinity receptors. Mast cells are also stimulated/activated by several cytokines, chemokines, hormones, such as corticotropin-releasing hormone (CRH), toxins and extreme external environmental changes [23,36,44,45].

Upon stimulation, mast cells secrete multiple biologically active mediators [46], some of which are preformed and stored in as many as 1000 secretory granules per cell, such as β-hexosaminidase (β-hex), heparin, histamine, tumor necrosis factor (TNF) and the serine proteases chymase and tryptase through rapid (1–5 min) degranulation by exocytosis [47]. Histamine and tryptase are the main mediators commonly associated with mast cells [48]. Tryptase is found in all mast cells, but unlike mucosal mast cells (MMCs), which contain only tryptase, connective tissue mast cells (CTMCs) contain both chymase and tryptase. Even though these proteases are considered to be stored in the same secretory granules, there is evidence that this may not necessarily be true. For instance, serum tryptase was not elevated in many patients with MCAS [28] or in cutaneous mastocytosis [49]. In one paper, it was shown that IgE-mediated degranulation of primary murine MMCs and CTMCs released phenotypically different extracellular vesicle (EV) populations depending on the stimulus [50]. In particular, unstimulated mast cells constitutively released CD9+ EVs, while degranulation was accompanied by the release of CD63+ EVs that contained different proteases [50].

Mast cells also release newly synthesized phospholipid products such as prostaglandin D_2_ (PGD_2_) and leukotrienes (LTs) [51,52,53], as well as numerous de novo synthesized protein mediators 6–24 h after stimulation such as interleukins [54], including interleukin-1beta (IL-1β) [55], IL-6 [45,56], IL-31 [57], IL-33 [55] and TNF [43].

Mast cells can secrete their numerous mediators [25,47,58] utilizing different signaling [11,59,60,61,62] and secretory [60,63,64] pathways sometimes referred to as the “secretome” [65]. The secretory pathways include degranulation by exocytosis, compound exocytosis, piecemeal degranulation, transgranulation, directed degranulation, vesicular (differential) release of mediators, extracellular nanovesicles (exosomes), nanotubules [66] and antibody-dependent “immunologic synapses for dedicated secretion” [67,68] (Table 1). The term “secretion” is used in this review to include both degranulation by exocytosis, which is the main means of secretion of granule-stored mediators [69], as well as differential release via which chemokines and cytokines are released without degranulation [59]. For instance, it was first reported that serotonin [45,52,56], and later, vascular endothelial growth factor (VEGF) [70] and IL-6 [45,56], could be secreted from mast cells without degranulation and without the release of histamine or tryptase [59]. It has also been reported that mast cells can release the content of individual secretory granules [71] or individual mediators without degranulation [52]. This process was distinct from “piecemeal degranulation” [72], granule-associated vesicle transport [63] or the release of extracellular vesicles [67,73,74,75,76,77,78].

Moreover, mast cell mediators could have autocrine actions affecting the expression of receptors or the overall reactivity of mast cells. For instance, mast cells can release the “alarmin” IL-33 themselves [55]. IL-33 then could stimulate mast cells via the activation of its own specific surface receptor ST2 and significantly increase the ability of substance P (SP) to stimulate secretion of VEGF [79,80], IL-31 [57], TNF [43] and IL-1β [55]. Mast cell-derived IL-1β or histamine could further stimulate the release of IL-1β from macrophages [81]. IL-1β could, in turn, stimulate mast cells to release IL-6, which was shown to stimulate mast cell proliferation [82]. The presence of the D816V-KIT mutation in mast cells was associated with constitutive release of IL-6 [83]. Serum levels of IL-6 were reported to be elevated in mastocytosis [84,85,86] and correlated with disease severity. Mast cells could also undergo directional degranulation and secretion of TNF and possibly other pro-inflammatory mediators into the bloodstream [87]. It is also important to note that mast cells exhibit different phenotypes including expression of different receptors depending on the tissue microenvironment [88]. Moreover, different receptors may interact and increase mast cell reactivity [89], as shown for FcεRI and MRGPRX2, which were reported to have an additive effect in stimulating degranulation of human skin mast cells [90]. 

IL-33 increased the expression of the SP receptor neurokinin-1 (NK-1), while SP increased expression of the IL-33 receptor ST2 [55]. SP also induced the expression of the receptor CRHR-1 for the key stress hormone CRH in human mast cells [91]. Instead, SP downregulated the expression of FcεRI in human mast cells [92]. CRH stimulated mast cells to release VEGF without degranulation, an action that was augmented by the peptide neurotensin (NT) [93]; during this process, CRH stimulated the expression of the NT receptor NT3, while NT stimulated the expression of CRHR-1 [94]. These findings could help explain why many atopic patients worsen dramatically after a major stressful episode [95,96].

Mast cell-derived mediators could also induce epigenetic effects as shown for tryptase, which could catalyze histone clipping [97] and could regulate modification of histones in mast cell leukemia cells [98]. The expression of Ten-eleven translocation-2 (TET2), an epigenetic regulator, was induced in response to the activation of mast cells [99,100]. Hence, mast cells are very dynamic cells that respond not only to external but also to innate stimuli. Such findings have prompted the re-evaluation of the secretory processes and their regulation in mast cells [101].

## 3. Regulation of Mediator Secretion from Mast Cells

Our understanding of the regulation of mediator release via the different modes of secretion and its regulation is still poorly understood. Even though the stimulus–response coupling pathway has been well delineated for activation of the high-affinity surface receptor for IgE (FcεRI) [42,102,103], and, more recently, of the low-affinity receptor for cationic peptides, Mas-Related G Protein-Coupled Receptor-X2 (MRGPRX2) [104,105,106,107,108], there is still a lack of understanding of the molecular events regulating secretion, whether by degranulation, selective release of mediators or any other mode of secretion (Table 1). The mode and extent of mast cell responsiveness ultimately depend on the interplay between stimulatory and inhibitory signaling pathways, such as CD300 [109,110] and Singlets [111], especially Siglec-7 [112], and the β subunit of FcεRI (FcεRIβ) [113].

### SNAREs and SNAPs

One possible mechanism of how mast cell secretion may be regulated could involve the Soluble NSF attachment protein receptors (SNAREs) and synaptosomal-associated proteins (SNAPs) discovered by Dr. J.E. Rothman, who was awarded the 2013 Nobel Prize in Physiology and Medicine for delineating the principles for secretory membrane fusion [114]. The existence of distinct secretory vesicle calcium-sensitive proteins responsible for “snapping” with corresponding proteins on the plasma membrane during secretion by exocytosis from mast cells had actually been proposed much earlier by one of the authors (TCT) in his doctoral thesis examination at Yale University in 1974, with the examiner being Dr. G. Palade, who had just received the 1974 Nobel Prize in Physiology and Medicine for his discovery that secreted proteins are carried from the endoplasmic reticulum (ER) to the cell surface in specialized compartments or transport vesicles. 

SNAREs [115,116,117] and synaptosomal-associated protein of 23 kDa (SNAP-23) [118,119,120,121,122,123] have been shown to be involved in mast cell secretion. In fact, there may be different mechanisms regulating exocytosis in mast cells [124], and mast cell distinct secretory granule subsets may be regulated by different SNARE isoforms [125] and different vesicle-associated membrane proteins (VAMPs), especially VAMP2- and VAMP8 [126,127].

Mast cells express Munc18-2, which interacts with SNARE syntaxin 2 or 3, as well as Munc18-3, which interacts with syntaxin 4. Munc18-2 was localized to secretory granules, whereas Munc18-3 was found on the plasma membrane. Increased expression of Munc18-2 inhibited IgE-triggered exocytosis, while increased expression of Munc18-3 had no effect. Upon stimulation, Munc18-2 redistributed on granules that were aligned along microtubules, but was excluded from F-actin ruffles, suggesting a role for Munc18-2 and the microtubule network in the regulation of secretion by degranulation in mast cells [128]. In addition, a number of so-called ‘adapters’ have been reported to regulate secretion from mast cells by binding multiple signaling proteins and localizing them to specific cellular compartments [40].

It Is of note that the degranulation of different mast cell vesicle subsets was differentially and selectively regulated by various polyphenols via interfering with two SNARE complexes, Syn (syntaxin) 4/SNAP-23/VAMP2 and Syn4/SNAP23/VAMP8 [129]. Similarly, polyphenols were shown to interfere with “zippering” of SNARES in the neuron [130]. The structure of the phenolic flavonol quercetin is somewhat similar to cromolyn [131] but is a more potent inhibitor of mast cells than cromolyn [132]. Quercetin inhibited rat mast cell degranulation [133,134], possibly via the inhibition of protein kinase C (PKC) [135,136], but it also induced the phosphorylation of moesin [136]. Quercetin also inhibited the release of pro-inflammatory cytokines [135], including IL-6 [134], from cultured human mast cells. The quercetin-related flavone luteolin and the luteolin analogue tetramethoxyluteolin were even more potent inhibitors of both the degranulation [137] as well as of release of TNF [43] and IL-1β [55] from human mast cells. The ability of flavonoids to inhibit mast cell secretion via phosphorylation of moesin led to conjectures about the design of more potent inhibitors [131].

In spite of the advances briefly outlined above, there is still no effective inhibitor of mediator secretion from mast cells. Antihistamines interfere with histamine binding to its receptors after it has been secreted. There has been considerable progress in developing drugs that block tyrosine kinases involved in mast cell proliferation [138].

## 4. Ezrin, Radixin and Moesin (ERM) Family of Proteins

Ezrin, radixin and moesin (ERMs) are fairly homologous proteins (73% amino acid identity) that link the actin cytoskeleton to the cytoplasmic tail of transmembrane proteins in the plasma membrane, thus regulating the formation of F-actin-based structures [139,140,141,142,143,144]. ERMs localize to cell surface protrusions such as microvilli, filopodia and cell–cell junctions. ERMs are critical for signal transduction from the cell surface into the cell. Given the high degree of homology and their co-expression to various degrees in many cell types, overlapping or even compensatory functions have been proposed.

**Figure 1:**
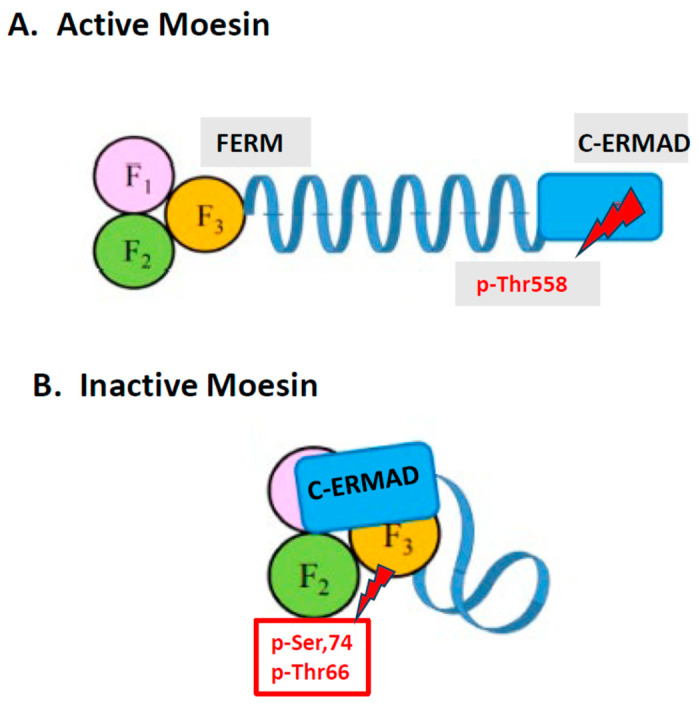
Diagrammatic representation of the active and inactive forms of moesin. Phosphorylation of moesin at Thr558 opens up actin-binding sites. In contrast, phosphorylation of moesin at Ser56/Thr66 changes the conformational structure of moesin so that Thr558 is no longer accessible to bind to actin, thus preventing secretion.

Ezrin was named after Ezra Cornell University where it was first isolated from microvilli in chicken intestinal epithelial cells, while radixin (from the Latin meaning root) was isolated from the adherens junctions of rat liver hepatocytes. Moesin (membrane-organizing extension spike protein) was isolated from smooth muscle cells of the bovine uterus. ERMs contain two functional domains connected through a long α-helix region (Figure 1A): the N-terminal FERM (band 4.1 protein-ERM) domain, which is critical for the function of the ERMs, and the C-terminal ERM association domain (C-ERMAD). The FERM domain is composed of three subdomains (F1, a ubiquitin-like domain; F2, with four α-helices; and F3, a pleckstrin homology domain). The FERM domain and the C-ERMAD can bind each other in a head-to-tail manner, leading to a closed/inactive conformation (Figure 1B).

**Figure 2:**
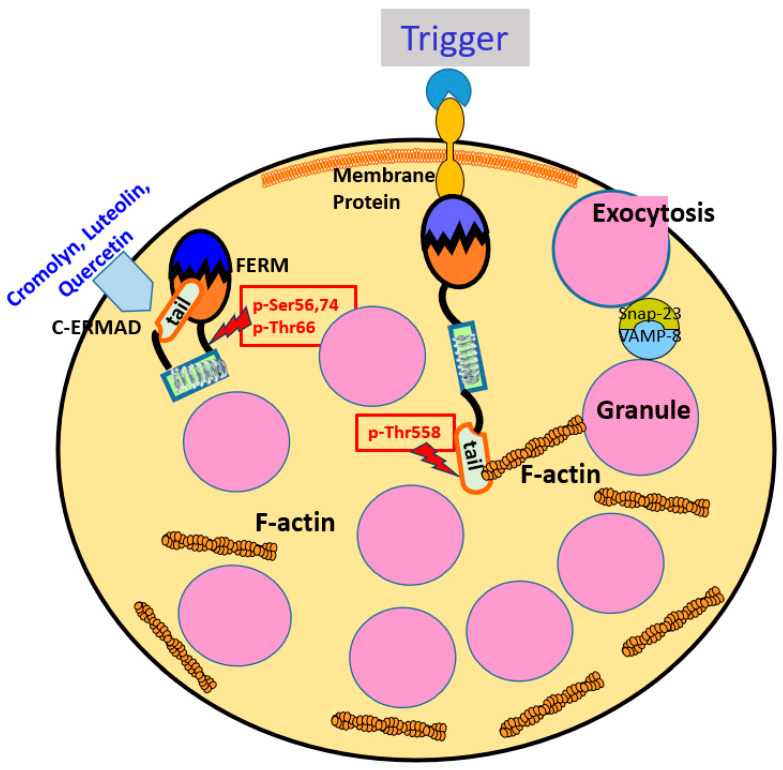
Moesin in Mast Cell Secretion. Diagrammatic representation of how differential phosphorylation of moesin could regulate secretion from mast cells. Phosphorylation of moesin at Thr558 in response to triggers opens up binding sites permitting granules to travel to the cell surface and secrete granule-stored mediators via degranulation. In contrast, phosphorylation of moesin at Ser56/Thr66 by cromolyn or flavonoids changes the conformational structure of moesin so that Thr558 is no longer accessible to bind to actin, thus preventing secretion.

The release of the C-ERMAD from the FERM domain is necessary for the activation of ERMs, unmasking their F-actin- and PM-binding sites. Activation of ERMs occurs first by phosphatidylinositol 4,5-bisphosphate (PIP2) binding to the N-terminus and changing the 3D structure exposing a C-terminal Threonine (Thr567 in ezrin, Thr564 in radixin and Thr558 in moesin) for phosphorylation [140,145] by the Rho family of GTPases (RhoA/Rac/Cdc42). This step transitions ERMs from a closed (inactive, Figure 1B) to an open (active, Figure 1A) conformation [146] that exposes the C-terminal F-actin-binding domain that cross-links plasma membrane proteins with actin filaments (Figure 2) [140,143,144,145,146].

### Moesin in Mast Cells

The expression of particular ERM members varies among different cells. Moesin is mainly expressed in endothelial cells, with ezrin in intestinal epithelial cells and radixin in hepatocytes. However, moesin is the most abundant ERM in leukocytes and mast cells, whereas ezrin is less expressed, and radixin is nearly absent [142].

Mast cells, like any other secretory cell, require the actin cytoskeleton [147] that is necessary for signal transduction and movement of secretory granules or vesicles destined for secretion to the cell surface. For instance, the aggregation of IgE bound to FcεRI by a multivalent antigen stimulates mast cell secretion and rapidly depolymerizes actin filaments, with the actin-severing protein cofilin being dephosphorylated several minutes after stimulation [148]. In contrast, the disaggregation of IgE terminates degranulation mediated by dephosphorylation of Syk associated with a decrease in intracellular Ca^2+^ concentration and rapid recovery of actin polymerization. Upon FcεRI stimulation, Dok-1 (downstream of tyrosine kinase 1) undergoes Tyr phosphorylation, which negatively regulates Ras/Erk signaling and subsequent secretion by inhibiting calcium influx and calcium-dependent disassembly of actin filaments [149]. It was previously shown that Rho GTPases regulate exocytosis and possibly secretory granule transport. One paper used live-cell imaging to analyze cytoskeleton assembly and secretory granule transport in real-time of mast cells or rat basophil cells (RBL-1) during antigen stimulation. This paper showed that granule transport to the cell periphery was coordinated by de novo microtubule formation and not F-actin since kinesore, which activates the microtubule motor kinesin-1 inhibited microtubule-granule association and significantly reduced degranulation [150]. However, how F-actin or microtubules communicate with secretory granules (or vesicles) and the plasma membrane is still not well understood. Knockdown of the unconventional long-tailed myosin (MYO1F), which localizes with cortical F-actin by short hairpin RNA, reduced human mast cell degranulation stimulated by both IgE and MRGPRX2, and was accompanied by reduced reassembly of the cortical actin ring and fewer secretory granules localized close to the cell surface [151]. Interestingly, MYO1F knockdown also resulted in fewer fissioned mitochondria and deficient mitochondria translocation to sites of degranulation by exocytosis [151]. Mitochondria fission was also reported to accompany secretion by degranulation, but not during secretion of de novo synthesized mediators from human mast cells stimulated by SP [18] and also in skin biopsies from patients with atopic dermatitis [152]. It was further shown that stimulation of mast cells resulted in extracellular secretion of mitochondrial DNA (mtDNA) that acted as an “innate pathogen” and triggered an autoinflammatory response. Increased levels of mtDNA have been reported in patients with COVID-19 [153,154,155,156], psoriasis [157], as well as in EVs from patients with myalgic encephalomyelitis/chronic fatigue syndrome (ME/CFS) [158] and from children with autism spectrum disorder (ASD), and in both cases, mtDNA activated cultured human microglia to secrete IL-1β [159].

The ability of the so-called “mast cell stabilizer” disodium cromoglycate (cromolyn) to inhibit secretion from rat mast cells in response to the cationic Compound 48/80 (C48/80) was shown to closely parallel the phosphorylation of a 78 kDa protein [135,160,161] on the *N-terminal Ser56, Ser74 and Thr66* residues (Figure 1B) [162]. We found that this protein was subsequently cloned from mast cells and was shown to be moesin [163], but we named it Mast Cell Degranulation Inhibitory Agent (MACEDONIA) [164]. It is important to note that phosphorylation of at least the *N-terminal Ser56/74 and Thr66* residues during inhibition is different to the well-known phosphorylation of C-ERMAD Thr558 associated with moesin activation. In support of the involvement of additional phosphorylation sites than Thr558, there is evidence that, at least in ezrin, Thr235 is phosphorylated by cyclin-dependent kinase 5 (CDK5) and cooperates with Thr576 for its full activation [165].

Using confocal microscopy and ultra cryo-immuno-electron microscopy to preserve the antigenicity of ERMs, it was shown that mast cells contain almost exclusively moesin (with a small amount of ezrin), which was critically localized primarily at the plasma membrane and filopodia, with less around secretory granules; it was further shown that cromolyn induced the clustering of moesin around secretory granules [163]. It was therefore hypothesized that conformational changes in moesin due to phosphorylation/dephosphorylation events could possibly regulate mast cell secretion via positional rearrangements with respect to the membrane/cytoskeleton [163]. It was further hypothesized that moesin could, in fact, serve a dual function depending on its phosphorylation pattern, which occurs after a trigger or an inhibitor interacts with the cell surface [131]. In other words, moesin phosphorylation at C-terminal Thr558 would switch moesin to its active form (Figure 1A) and permit mast secretory granules to move to the surface, fuse with the plasma membrane and undergo exocytosis (Figure 2). In contrast, phosphorylation of N-terminal Ser/Thr sites would switch moesin to its inactive state (Figure 1B) resulting in either (a) the prevention of phosphorylation of Thr558 and moesin activation, (b) the interaction with secretory granules preventing them from moving to the cell surface or (c) affecting the structure of the cell cortex and block secretion indirectly (Figure 2). However, it remains unknown how the phosphorylation of moesin at different sites affects secretion from mast cells in response to different triggers, and how phosphorylation at the N-terminal sites mechanistically leads to the inhibition of mast cell secretion. Moreover, it is not presently known if phosphorylation of moesin may affect modes of secretion other than degranulation by exocytosis. One paper identified a number of Ser/Thr-phosphorylated proteins in activated mast cells, including moesin, but these were involved in different processes such as metabolism and cell structure [166]. Even though ezrin has been mostly discussed for its involvement in cancer [167], it is not known if ezrin could compensate for moesin should the latter be absent or “incapacitated” in mast cells. In fact, ezrin, has been implicated in asthma [168]. The phosphorylation of ezrin at Thr567 was associated with trophoblast motility [169].

Interestingly, moesin knock-out mice were shown to have lymphopenia [170], but mast cell numbers were apparently intact; however, the authors did not investigate mast cell secretion [170]. One X-linked moesin-associated immunodeficiency (X-MAID) has been identified and is characterized by a primary immunodeficiency associated with severe lymphopenia leading to recurrent infections. X-MAID is caused by a single-point mutation leading to a R171W amino acid change in moesin (moesinR171W) [171]. In fact, a mouse model with global expression of moesinR171W exhibited lymphopenia, but it was still characterized by systemic inflammation [171].

The phosphorylation of moesin has also been studied in other secretory systems. Moesin was shown to be phosphorylated at Thr558 within seconds of thrombin-induced activation of platelets [172,173]. Instead, the tyrosine phosphorylation of moesin was reported during the activation of platelets with arachidonic acid [174]. These phosphorylation patterns are reversed by protein phosphatase 2C, which inactivates the F-actin-binding site of activated platelets [175]. Phosphorylation at Thr558 was also reported in activated RAW264.7 macrophages [176]. ERM proteins have been shown to be involved in T-cell polarization and immune synapse formation [177]. It is interesting that anti-moesin autoantibodies were isolated from patients with aplastic anemia [178] and autoimmune vasculitis [179]. However, the significance of these autoantibodies is not apparent, nor is their potential presence in patients with allergies and inflammatory disorders.

## 5. Mast Cells and Moesin in Neuroinflammation

**Figure 3:**
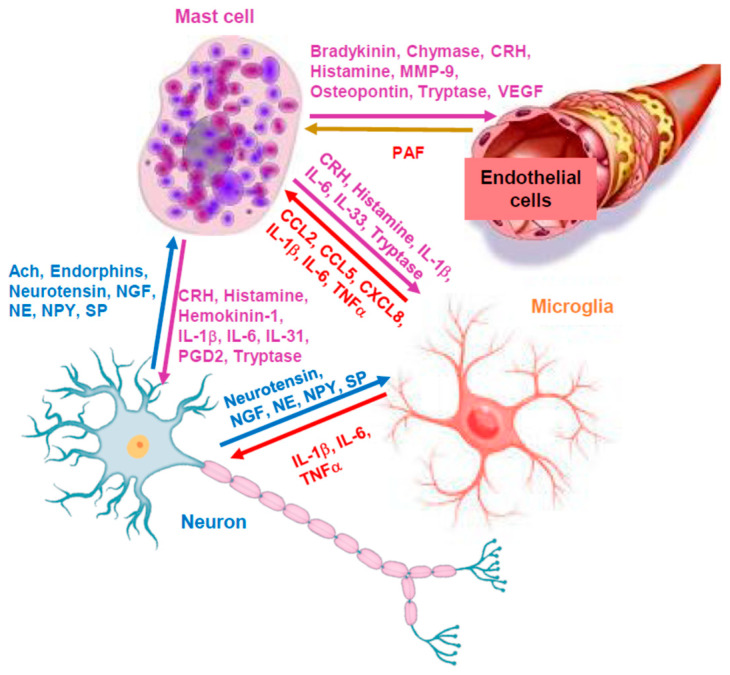
Diagrammatic representation of the key role of mast cells in neuroinflammation. Mediators released from mast cells can stimulate endothelial cells, microglia and neurons directly to promote inflammation; in turn, molecules secreted from the other cells can stimulate mast cells, thus further promoting neuroinflammation. ERMs could regulate secretion of mediators from mast cells, but also from the other cell types involved. Ach = acetylcholine; CRH = corticotropin-releasing hormone; MMP9 = metalloproteinase-9; NGF = nerve growth factor; NE = norepinephrine; NPY = neuropeptide Y; NT = neurotensin; PAF = platelet activating factor; PGD2 = prostaglandin D2; SP = substance P; VEGF = vascular endothelial growth factor.

Mast cells communicate with microglia [180,181] and can activate them [181,182,183,184] via the release of mediators such as histamine [185] and tryptase [186], leading to neuroinflammation [180,182] (Figure 3). The activation of mast cells and microglia in the brain [187] could affect neurodevelopment [188], resulting in neuronal apoptosis [189], and lead to cognitive dysfunction [189]. In fact, the activation of mast cells and microglia has been linked to the pathogenesis of autism spectrum disorder (ASD) [190,191,192,193,194], neurodegenerative diseases [35,195] and traumatic brain injury (TBI) [24,196]. It is, therefore, of interest that moesin has been reported to be involved in the activation of microglia [197]. Moreover, the moesin pseudogene 1 antisense (MSNP1AS) gene was shown to decrease the number and length of neurites, reduce neural viability and promote apoptosis via the inhibition of moesin protein expression, while moesin improved social interactions and reduced repetitive behaviors in BTBR mice [198].

Moreover, one paper reported that ezrin, radixin and moesin had distinct roles of in maintaining the plasma membrane integrity and functions of the blood–brain barrier (BBB) transporters [199], which is important because mast cells can regulate the permeability of the BBB [200], the disruption of which has been implicated in ASD [201], in Alzheimer’s disease [33] and in neuro-COVID-19 [202]. ERMs could regulate the secretion of mediators from mast cells but also from the other cell types involved in neuroinflammation.

In this context, it is relevant that flavonoids could have anti-inflammatory [34,203,204,205,206,207,208,209] and neuroprotective effects [210], as well as reduce cognitive dysfunction [211,212,213,214,215], especially brain fog [216,217,218]. In particular, luteolin inhibited both microglia [219,220,221] and mast cells [222,223]. One formulation containing liposomal luteolin in olive pomace (fruit) oil (NeuroProtek^®^) resulted in significant improvement in children with ASD [224], with a concomitant decrease in serum inflammatory markers [225]. Other papers reported the beneficial actions of luteolin in Long-COVID-19-associated brain fog [216,226] and neurotrauma [207].

## 6. Conclusions

The studies reviewed indicate that the pattern of phosphorylation and localization of moesin may be important in the regulation of exocytotic secretion of at least secretory granule-associated mediators such as histamine, TNF and tryptase.

It will be important to investigate the expression of total and phosphorylated moesin in human mast cells of different degrees of reactivity/types, such as the leukemic human mast cell line-1 (HMC-1), the Laboratory of allergic diseases-2 (LAD2) and LADR mast cells [227], as well as primary human umbilical cord blood-derived cultured mast cells (hCBMCs), mast cells developed from pluripotent stem cells [228,229,230], but also mast cells from cutaneous mastocytosis or urticaria lesions. Other future studies should investigate whether the knockdown of moesin using small interfering ribonucleic acid (siRNA) would affect the extent of secretion or interfere with the ability of cromolyn or flavonoids to inhibit mast cell secretion. Additionally, studies should also investigate which specific sites are phosphorylated in response to triggers or inhibitors of either the degranulation or differential release of select mediators using trypsin-digested moesin peptides analyzed via mass spectrometry and validated with site-specific phospho-antibodies and point mutant analysis.

It will also be important to investigate the possible presence of some innate molecule(s) or identify novel molecules that could keep moesin in its inactive state, for the development of new effective anti-allergic and anti-inflammatory drugs.

## Figures and Tables

**Table 1:** Different modes of secretion of mediators from mast cells.

Degranulation (exocytosis)
Compound exocytosis
Piecemeal degranulation
Transgranulation
Directed degranulation
Vesicular (differential) release of mediators
Extracellular nanovesicles (exosomes)
Nanotubules
Immunologic synapses
